# Laser-induced graphene electrochemical sensor for quantitative detection of phytotoxic aluminum ions (Al^3+^) in soils extracts

**DOI:** 10.1038/s41598-024-56212-0

**Published:** 2024-03-08

**Authors:** Vanessa Reyes-Loaiza, Jhonattan De La Roche, Erick Hernandez-Renjifo, Orlando Idárraga, Mayesse Da Silva, Drochss P. Valencia, Thaura Ghneim-Herrera, Andres Jaramillo-Botero

**Affiliations:** 1 iOmicas Research Institute, Pontificia Universidad Javeriana, Cali, Valle del Cauca 760031 Colombia; 2 Multifunctional Landscapes, Alliance Bioversity-CIAT, Cali-Palmira, Valle del Cauca 763537 Colombia; 3 Department of Natural and Exact Sciences, Universidad del Valle, Cali, Valle del Cauca 760031 Colombia; 4 Department of Biological Sciences, Universidad ICESI, Cali, Valle del Cauca 760031 Colombia; 5 Chemistry and Chemical Engineering Division, California Institute of Technology, 1200 E California Blvd, Mail Code 139-74, Pasadena, CA 91125 USA

**Keywords:** Electrochemistry, Graphene, Sensors and biosensors

## Abstract

Aluminum in its Al^3+^ form is a metal that inhibits plant growth, especially in acidic soils (pH < 5.5). Rapid and accurate quantitative detection of Al^3+^ in agricultural soils is critical for the timely implementation of remediation strategies. However, detecting metal ions requires time-consuming preparation of samples, using expensive instrumentation and non-portable spectroscopic techniques. As an alternative, electrochemical sensors offer a cost-effective and minimally invasive approach for in situ quantification of metal ions. Here, we developed and validated an electrochemical sensor based on bismuth-modified laser-induced graphene (LIG) electrodes for Al^3+^ quantitative detection in a range relevant to agriculture (1–300 ppm). Our results show a linear Al^3+^ detection range of 1.07–300 ppm with a variation coefficient of 5.3%, even in the presence of other metal ions (Pb^2+^, Cd^2+^, and Cu^2+^). The sensor offers a limit of detection (LOD) of 0.34 ppm and a limit of quantification (LOQ) of 1.07 ppm. We compared its accuracy for soil samples with pH < 4.8 to within 89–98% of spectroscopic methods (ICP-OES) and potentiometric titration. This technology's portability, easy to use, and cost-effectiveness make it a promising candidate for in situ quantification and remediation of Al^3+^ in agricultural soils and other complex matrices.

## Introduction

Aluminum is one of the most abundant metallic elements on Earth, and it is found in the form of Al_2_O_3_ in over 15% of the Earth’s crust^1^. Its Al^3+^ form, prevalent in dry soils, is phytotoxic because it inhibits nutrient uptake by plant roots and hinders their growth. Its capacity to displace nutrient ions in cation exchange sites and bind to the root elongation zone, and its bioavailability in acidic soils (pH < 5.5), make it one of the main limiting factors in crop productivity^1,2^.

The advent of sensing technologies for rapidly detecting this ion in phytotoxic concentrations (> 2 ppm) provides a starting point for remediation to salvage the productivity of crops, particularly in acidic agricultural lands^3^. Furthermore, these technologies open the door to implementing new strategies to improve crop selection and optimize breeding^4^. However, the detection of trace metals has primarily been performed using spectroscopic methods, including inductively coupled plasma via mass or optical emission spectrometry (ICP-MS/OES), that require expensive instruments, materials, and qualified operators^5^. Therefore, these methods are not suitable for routine analysis of multiple samples^6^, which severely limits their application for widespread use in agriculture.

Colorimetric/fluorescence^7–9^ and electrochemical^5,10,11^ systems are considered cost-effective alternatives for identifying and quantifying metal ions in various matrices. Selective colorimetric and fluorescent probes enable fast visual identification of analytes. Still, in-the-field quantification remains a challenge. Recent studies demonstrate the use of carbon-based nanostructured electrodes for the electrochemical detection of metal ions with high reproducibility, sensitivity, and selectivity^12–15^. Among the carbon nanostructures used to fabricate these sensors, Laser-Induced Graphene (LIG) has most recently garnered attention for its simple manufacture, low cost, biocompatibility^16^, and potential for increased surface area, trough surface treatments that can enhance sensitivity and intensify the signals of the analyte of interest^17^. LIG electrodes have been shown to work for gas sensors^18^, biosensors^19,20^, among others^16,21–23^, across a wide range of applications in healthcare, environmental monitoring, and quality control in manufacturing.

Recent studies have reported the feasibility of LIG-based electrochemical sensors for detecting metal ions such as Cd^2+^ and Pb^2+^^24,25^. However, this technology requires understanding the nature and behavior of the graphene-based substrate material for each application^26^, since detecting metal ions with LIG electrodes depends on the material’s functionalization and the electrochemical technique used. Bismuth-based electrodes are ideal for heavy metal ion sensing due to their ability to electrodeposit elements on their surface through the formation of intermetallic compounds^27^. Additionally, bismuth (Bi) exhibits very low toxicity and possesses suitable electrochemical properties, including low background currents and the ability to separate intermetallic compounds^27–30^. Electrochemical techniques, such as square wave voltammetry represent highly sensitive detection methods for metal ions. This is due to their involvement of a preconcentration step and the continuous adsorption and desorption of the metal ions^31,32^. Prior studies using bismuth-functionalized electrodes and square wave voltammetry (SWV) have shown promising results in metal ion detection. Among these, Yi et al^33^. and Jeong et al^25^. successfully quantified Cd^2+^ in real water samples. Additionally, investigations carried out by our research group showcased the feasibility of this method in detecting trace amounts of Al^3+^ in an experimental solution^34^.

Detecting metals in foods or food sources requires sensitivities in parts per billion (ppb) concentrations when accumulation of trace amounts pose an immediate risk to human health^35^. This is the case of Cadmium (Cd), Mercury (Hg), Lead (Pb), and other heavy metals^36–38^. Human exposure to aluminum, on the other hand, is unavoidable in today’s world. Although chronic buildup of biologically reactive aluminum can lead to serious health problems, it is rarely acutely toxic^39^. However, Al^3+^ can be phytotoxic for plants when its concentration exceeds 2–3 ppm in soil^40,41^, including tolerant crops like rice and maize, where concentrations around 40–80 ppm of this ion have been associated with decreased biomass production in these species^42–44^.

While various electrochemical-based sensors for specific Al^3+^ detection have been developed, they primarily operate within a range designed to determine trace amounts of this metal ion, mainly within the low ppb range^34,45–47^. Unfortunately, the critical phytotoxic threshold to effectively monitor the Al3 + content in agricultural soils starts at 0.3 ppm and extends beyond > 200 ppm^48,49^. None of the existing technologies cover this range. Here, we report for the first time, the development, characterization, and validation of a cost-effective, discardable electrochemical sensor based on LIG for accurately detecting and quantifying Al^3+^ in soils within the relevant concentration range for phytotoxicity in plants (ppm). The remainder of this document describes the materials and methods used in the fabrication of our disposable LIG-based electrochemical Al^3+^ sensors, the electrochemical methods used to characterize Al^3+^ concentrations using this sensor, the morphological and chemical characterization of the functionalized sensing electrode, and its Al^3+^ sensitivity and selectivity in the presence of other metal ions (Pb^2+^, Cd^2+^, and Cu^2+^), and the validation of the sensor using reference soil samples. We close by listing the key contributions and potential applications of this technology.

## Materials and methods

### Reagents

The standard stock solution of Al (III) 1000 mg L^−1^ was purchased from MOL LABS. The other reagents, including Pb (II), Cd (II) and Cu (II) (1000 mg L^−1^) standards, were purchased from SIGMA (ref: 41,318; 36,379). A stock Bi (III) solution 0.2 mol L^−1^ was prepared by adding Bi(NO_3_)_3_·5H_2_0 into acetate solution (sodium acetate + acetic acid 0.1 mol L^−1^) at pH 3.70. Working solutions were prepared in a concentration range from 1 to 300 ppm by diluting the stocks in acetate solution at 0.1 mol L^−1^ (pH 3.70) containing the Bi (III) solution at 0.1 mol L^−1^. The Ferri solution used for electrochemical characterization consisted of a solution of potassium hexacyanoferrate (III) [K_3_Fe (CN)_6_] 2 mmol L^−1^ (Alfa Aesar, ref: 33,357) with H_2_SO_4_ 0.18 mol L^−1^. All solutions were prepared with ultrapure water obtained from Thermo Fisher Scientific Barnstead SMART2PURE water system, with conductivity 18.2 Ω. For LIG fabrication, we used Polyester Diagnostic Tape (PT) (3 M™, ref: 9964) as a substrate, 50 mm thickness Polyimide (PI) film (Shijiazhuang Dadao Packaging Materials Co., Ltd), PELCO® Conductive Silver Paint (Ted Pella Inc, ref: 16,062) for all electrode contact points, and Silver/Chloride-silver (Ag/AgCl) ink (DuPont™, ref: 5874) for the pseudo-reference electrode.

### Fabrication, cleaning, and in situ preparation of LIG-based electrochemical sensors.

**Figure 1:**
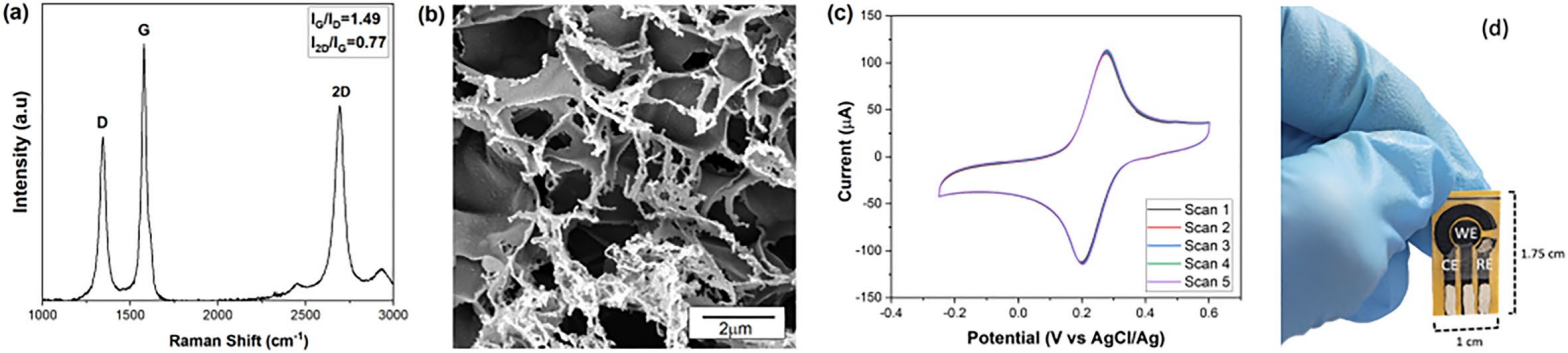
Characterization of a LIG electrode. (**a)** Raman spectra (**b)** SEM image at 40kx magnifications, (**c)** voltammogram after 5 scans with 2 mmol L^−1^ K_3_Fe (CN)_6_ + H_2_SO_4_ 0.1 mol L ^−1^, and (**d)** image of LIG-based 3-electrode electrochemical sensor cell. The data shown correspond to a representative response from a single electrode.

LIG-based planar electrochemical cells with three electrodes (working, reference and counter) were fabricated from PI (see Figure S1), using an FSL Muse 3D (Full spectrum laser) equipped with a 45 W CO_2_ laser (*λ* = 10.6 μm). The working electrode was set to a diameter of 4 mm. Lasing was performed at 9 W of power, at a laser head speed of 19.5 cm/s, using raster mode, and a constant laser lens focal distance to the polyimide sheets of 4.65 mm. These parameters were chosen, taking into account our earlier laser optimization studies^50^, to achieve the highest quality LIG suitable for sensing applications of this nature. The use of Ag/AgCl for the pseudo-reference electrode (RE) material is known to provide a consistent and reproducible potential^51,52^ Thus, we coated the pseudo-reference electrode (RE) with Ag–AgCl ink, and used an Ag ink to improve the mechanical stability and conductivity for all electrode contacts, as depicted in Fig. 1d. To improve the chemical potential stability of the pseudo-RE, we incubated it overnight with 100 ml of KCl solution at 3 mol L^−1^. This is the standard background solution for preserving the commercial Ag/AgCl electrode^53^. Excess KCl was washed off the RE with Type I water before being cleaned with successive Cyclic Voltammetry (CV) scans using a 2 mmol L^−1^ [K_3_Fe (CN)_6_] + H_2_SO_4_ 0.18 mol L^−1^ solution over a potential range of − 0.25–0.6 V and a rate of 0.10 V s^−1^. Several CV scans are often conducted to eliminate any adsorbed species from the electrode surface^54^. This procedure was repeated until a smooth voltammogram form was obtained (up to six scans, see Figure S1). Five additional cycles were performed to confirm the sensor’s electrochemical repeatability (Fig. 1c). Then 40 μl of the working solution, containing both Bi (III) and the sample, was deposited and preconcentrated onto the WE surface for anodic stripping analysis. This resulted in *in-situ* electrodeposition of Bi (III), which reduces the complexity and time for modifying the WE, compared to a priori functionalization^34,55^. In the case of incremental measurements we conducted an electrochemical desorption step right after the stripping step and before adding the subsequent working solution over the surface of the same WE. This by applying an oxidizing potential of + 1.6 V for 60 s to remove the excess of Bi-Al^3+^ using the same working solution.

### Electrochemical detection of Al^3+^

Electrochemical experiments were conducted via Square Wave Anodic Stripping Voltammetry (SWASV), with a pre-concentration for measuring trace metals^56^. The procedure consists of preconcentrating the WE with the Bi^3+^ and Al^3+^ mixture for 60 s at a potential of − 1.40 V, followed by successive stripping potential cycles between − 2.00 to − 0.50 V, at 2 mV step potential, using an SW amplitude of 20 mV and a frequency of 25 Hz. These parameters were selected based on our previous research findings^34^, and also considering relevant studies, such as the work by Jeong et al^25^. and Anastasiadou et al^56^. Each stripping current (*I*_p_) (Figure S2) was recorded to build the calibration curve for Al^3+^ quantification over eleven different aluminum concentrations (1, 5, 10, 20, 40, 70, 100, 125, 200, 225, and 300 ppm). A different set of electrodes was used per concentration. On a single sensor cell, another calibration curve was constructed for incremental depositions over the same range intervals (Figure S3). For interference analysis, we added Pb^2+^, Cd^2+^, and Cu^2+^ ions to each sample at the same Al^3+^ concentrations. Then 40 μl of each sample was drop-casted on the WE surface before performing electrochemical characterization. We confirmed the stability over time (longevity) of the electrochemical sensor’s response to Al^3+^, by characterizing samples with 1, 100, and 200 ppm Al^3+^ concentrations using three different electrodes, after 1, 15 and 30 days of fabrication. (Figure S4). All electrochemical tests were performed using a reference potentiostat–galvanostat (Metrohm Autolab model PGSTAT302N, with the software NOVA 2.1).

### Morphological, chemical and structural characterization

To study the changes in the morphology of the bare and modified WEs, we used a Tescan Clara Field Emission Scanning Electron Microscope (FE-SEM) equipped with energy-dispersive electron X-ray (EDX) analysis system for chemical identification, and Thermo Fisher DXR SmartRaman spectrometer, with a 532 nm laser, 6 mW power in a range between 1000 and 3000 cm^−1^. Porosity was estimated via digital image analysis using the imageJ image processing software^57^ from three different images of the WE, obtained with a low-energy backscattered electron (LE-BSE) detector at 40 Kx magnifications, and an acceleration voltage of 10 kV.

### Al^3+^ extraction procedure from soil samples

The analytical services laboratory from the CIAT-Bioversity Alliance supplied soil samples. Three samples were collected from the Cauca, Carimagua, and Santander regions in Colombia. The last two are considered main cocoa-producing regions, which are known to have dry and acidic soils, and are used as an internal control for determining exchangeable Al^3+^ in soils through the volumetric standard method, potentiometric titration, and 1 mol L ^−1^ KCl extraction^58^. The soil sample from Cauca was collected near an industrial zone, from a local sugarcane mill plantation. The pH was measured at 3.70, 4.10 and 4.50, via potentiometry, for Cauca, Carimagua, and Santander respectively. A reference soil sample from WEPAL (Wageningen Evaluating Programmes for Analytical Laboratories) ISE-2020-3-3.1, with a higher pH (5.40), was also measured. We followed the extraction protocols reported by Wang et al. Zhao et al. and Krasnodębska-Oskęga & Kowalska with slight modifications^59–61^. We added 40 ml of acetic acid at 0.43 mol L^−1^ at pH 2.62–1 g of the soil sample in a 50 ml propylene tube. The mixture was mechanically shaken for 60 min and then allowed to settle for another two minutes. The mixture was filtered using a plastic funnel with filter paper attached to a new 50 ml tube. The resulting filtrate was adjusted with NaOH 5 mol L^−1^ to a pH of 3.70 and used as a direct substrate for subsequent analyses. For further validation, Al^3+^ content in the samples was also determined by Inductively Coupled Plasma with Optical Emission Spectroscopy (ICP-OES) Series iCAP™ 7000 and a Flame Atomic Absorption Thermo Scientific S4.

### Al^3+^ diagram species

To study the behavior of Al^3+^ metal ions in aqueous acetate solution within the pH range considered. We simulated the species distribution diagrams (Figure S5a) using the free software MEDUSA (Windows interface) and HYDRA (database manager) developed by Puigdomenech. The resulting simulations agreed with speciation diagrams described in the literature for similar systems^62–66^. These curves were then used for pH correction in the determination of Al^3+^ in soil extracts with the sensor developed in this work. To create this species diagram, we used a total acetate ion concentration of 0.100 mol L^−1^ and Al^3+^ ion concentration of 10 ppm. Other species diagrams have also been made for other Al^3+^ concentrations, but the percentage of free Al^3+^ is always similar.

Based on the species diagram, we constructed (Figure S5b), which illustrates the overestimation of Al^3+^ at pH values above 3.70 and the underestimation at lower pH values. To obtain these estimates, we assumed that the measurement at pH 3.70 has 100% accuracy. To obtain the values at other pH values, we divided the measurement obtained at pH 3.70 by the percentage of underestimation or overestimation. Figure S5a show that detecting Al^3+^ at pH above 4.80 will be difficult due to other masking aluminum species. Therefore, pH values up to 4.80 are considered optimal for quantifying Al^3+^ using the technology reported here.

## Results and discussion

### Morphological and electrochemical characterization of bare LIG electrodes

Structural characterization of the LIG material in our electrodes was performed using Raman spectroscopy. Figure 1a shows representative Raman spectra of our LIG, in which bands D ($\sim$1350 cm^−1^), G ($\sim$1580 cm^−1^) and 2D ($\sim$2700 cm^−1^) indicate the presence of multilayered graphene scales (I_2D_/I_G_ ≈ 0.77), with a proportionally-low concentration of disorder and a small average inter-defect distance (I_D_/I_G_ ≈ 0.67)^67,68^. The estimated in-plane size of the graphene scale crystallites (*L*_*a*_) is 28.6 nm, obtained from ${L}_{a}[nm]=(2.4\times {10}^{-10}){\lambda }^{4}({I}_{G}/{I}_{D})$^69^, where *λ* [nm] is the radiation wavelength that induces Raman scattering. Figure 1b shows an SEM image of the morphology of one WE, including laminar graphene sheets, pores, and fibers at the edges of the sheets.

Electrochemical characterization of each LIG electrode was performed by a series of five CV scans, with 2 mmol L ^−1^ K_3_Fe (CN)_6_ + H_2_SO_4_ 0.1 mol L ^−1^ and a potential window of − 0.25–0.6 V. An acidic medium was chosen to improve the electron transfer between the LIG surface and the analyte of interest, as reported by Saveant’s group^70,71^. A representative CV from one WE are shown in Fig. 1c. This shows that the redox behavior of K_3_Fe(CN)_6_ over the LIG electrode surface was a chemically and electrochemically reversible process, with a peak-to-peak separation (∆E_p_) of 75 mV and an anodic to cathodic peak current ratio (Ip_a_/Ip_c_) close to 1^15,54,72^. This validates our LIG-WE, providing a suitable potential window and current range to perform further electrochemical analysis.

### Working electrode surface functionalization for Al3+ detection

**Figure 2:**
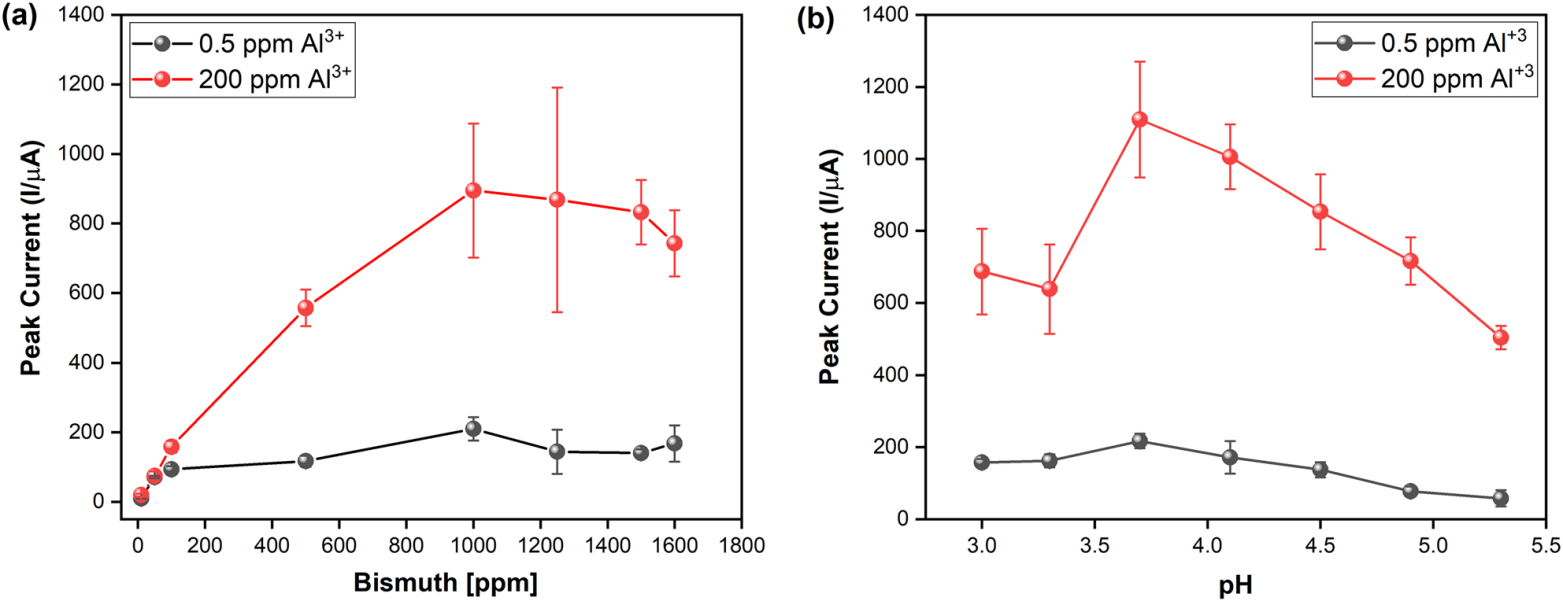
Optimization of chemical parameters for Al^3+^ identification and quantification. (**a)** Bi concentration versus peak current. (**b)** pH value of the buffer solution versus peak current. Each point corresponds to the mean, and the error bars to the standard deviation obtained from three independent electrodes.

Metal ions detection by SWASV with in situ bismuth deposition is a method that depends on Bi (III) concentration and the pH of the solution^30,73–75^. These parameters are optimized to obtain the highest stripping peak current in the SWASV for the analyte under study^25,76^. To achieve the highest sensitivity, we determined the solution's optimal Bi concentration and pH for Al^3+^ detection. For the Bi (III) analysis, we used SWASV to measure the peak current for Al^3+^ while increasing the concentration of Bi from 10 to 1600 ppm, as shown in Fig. 2a. Measurements were made independently for a low (0.5 ppm) and a high (200 ppm) concentration of Al^3+^. The highest peak current in both cases was 1000 ppm of Bi, consistent with previous studies on other metal ions, such as Cd^2+^ and Pb^2+^. This confirmed that the best stripping response is obtained at a Bi concentration five to 10 times higher than that of the analyte in solution^25,73,77^. After 1000 ppm Bi, the peak current starts to decrease, which indicates the formation of a Bi film and anchor site saturation for this species on the WE. This limits the mass transfer for Al^3+^ diffusion in the electrochemical system^25,73^, and affects the reproducibility in deposition kinetics dynamics of the analyte over the WE surface, which explains the increased error bars at the saturation point around 1200 ppm Bi. Consequently, we set the maximum Bi concentration at 1000 ppm.

We also measured the peak current of two concentrations (low and high) of Al^3+^, while varying the pH of the acetate solution from 3.0 to 5.3 (Fig. 2b). The highest peak current for Al^3+^ concentrations was obtained at a pH of 3.7. Below this value, we see a drop in peak current associated with the hydrogen evolution reaction (HER) that occurs on the WE surface, which limits the deposition of the target ion^78–80^. Above a pH of 3.7, the decrease in peak current can be explained by hydrolysis reactions of Al^3+^^15^ and the formation of metal hydroxides as evidenced in the speciation diagram for this ion, where the formation of Al(OH)_2_^+^ species starts to increase at pH 4^49,81^. Therefore, we set the sensing solution pH at 3.7.

### Incremental and one-time quantification of Al^3+^ using our LIG-based sensor

**Figure 3:**
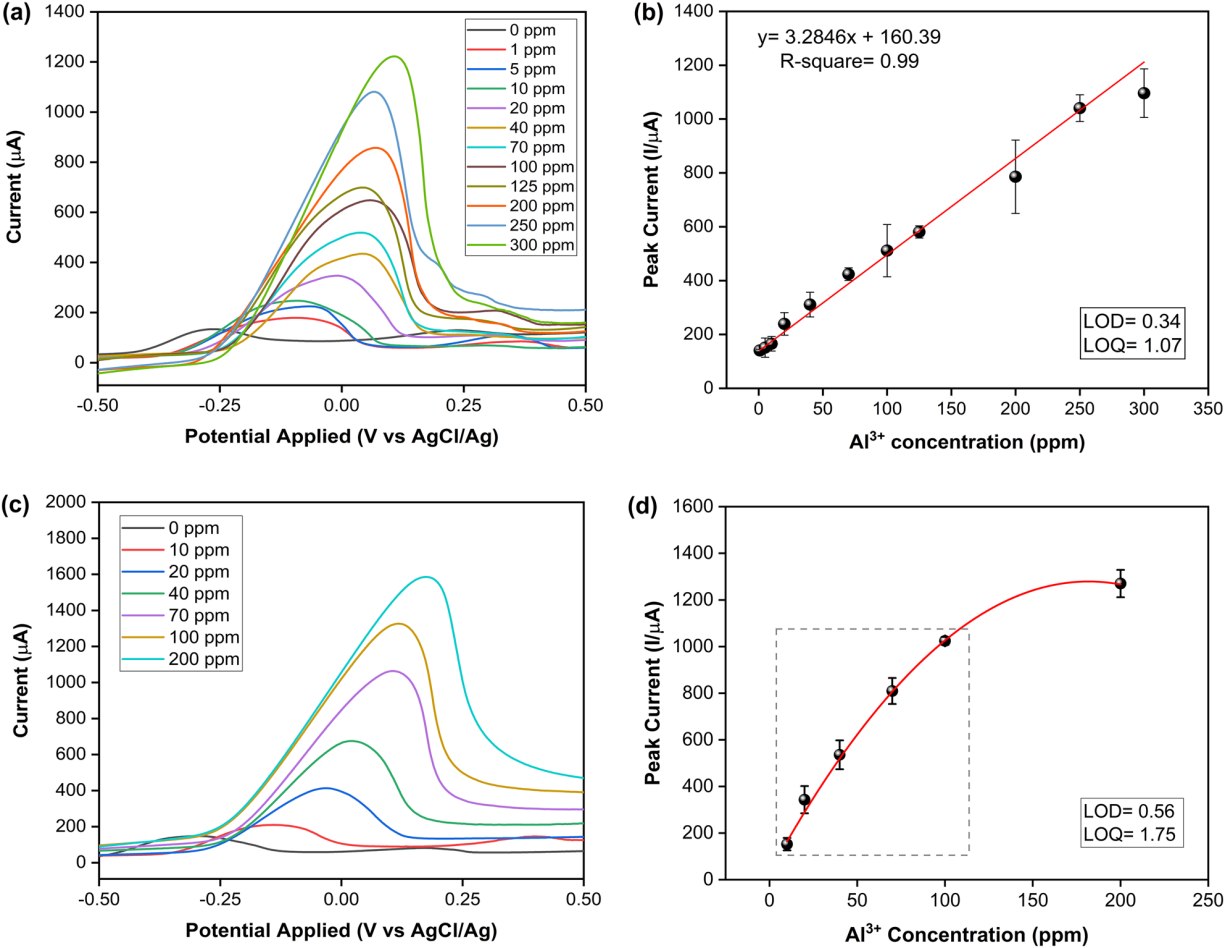
SWASV for detection of Al^3+^ on an LIG set of electrodes in acetate buffer 0.01 mol L ^−1^ (Acetic acid/ Sodium acetate) pH 3.7 and bismuth 1000 ppm. (**a)** SWASV curves for different concentrations of Al^3+^, each obtained using a different set of electrodes (one-time measurements). (**b)** Calibration curve obtained from the current peaks of the SWASV in Fig. 3a, (**c)** SWASV curves for different concentrations of Al^3+^ obtained from incremental concentrations of Al^3+^ on a single WE. **d** Calibration curve obtained from the current peaks of the SWASV in Fig. 3c. The gray dashed-line delimited area indicates a close to linear region up to 100 ppm.

We performed quantification analysis of Al^3+^ samples deposited during in situ Bi preparation with SWASV as described previously. The results are shown in Fig. 3a, where the peak current for Al^3+^ in solution was 1200 µA at around 0.10 V above a concentration of 100 ppm. Using the data from this curve, we constructed a linear calibration curve (with an R^2^ fit of 0.99) as shown in Fig. 3b. Each point in the figure corresponds to the average of at least three independent measurements, and the error bars were calculated using the standard deviation. It is important to note that a different LIG-based sensor was used for each case to ensure accuracy and reproducibility of the results.

The peak form shown in Fig. 3a is a typical response to electrochemical adsorption processes on the electrode surface^82^. As described previously^34^, the relationship between the peak current and the number of species adsorbed on the surface per unit volume (Γ*) can be expressed by equation, ${I}_{p}=({n}^{2}{F}^{2}/4RT)vA{\Gamma }^{*},$ where I_p_ is the peak current, *n* is the number of electrons transferred, *F* is the Faraday constant, *R* is the gas constant in J mol^–1^ K ^–1^, *T* is the temperature, *v* is the scan rate, *A* is the area of the electrode. Thus, higher peak currents correspond to a higher number of species absorbed on the surface. These results accurately reflect the relationship between peak current and the concentration of Al^3+^ deposited on the surface of the WE.

To investigate the capability of a single LIG-based sensor for quantifying different concentrations of Al^3+^ we performed stepwise deposits of aluminum on the WE, from 1 to 200 ppm (intervals listed previously). Figure 3a and c show that SWV stripping response increases as a function of Al^3+^ concentration, with the potential shifting to more positive values as the concentration increases. The change in the electrode response is due to the co-precipitation of bismuth and aluminum atoms. The concentration ratio of bismuth to aluminum ions changes the electron dynamics across the interface, primarily due to an increased film thickness. The bismuth film modifies the WE, and contributes to a well-defined, undistorted, and highly reproducible stripping response, with high-resolution neighboring peaks, high hydrogen evolution, a wide linear dynamic range, and a good signal-to-background characteristic, as explained by Wang^83^. The current peak shifts to more oxidative potentials for increasing film thicknesses are explained by the Hubbard model, i.e. more time is required to reduce the film of electroactive species^84^. This explains the changes observed between the response recorded for the baseline (only bismuth) and each of the increasing concentrations of Al^3+^ in the working solution, for both cases, one-time (Fig. 3a) and incremental measurements (Fig. 3c), with the latter exhibiting a more pronounced shift due to the cumulative deposition of both bismuth and Al^3+^ over the same WE surface.

**Figure 4:**
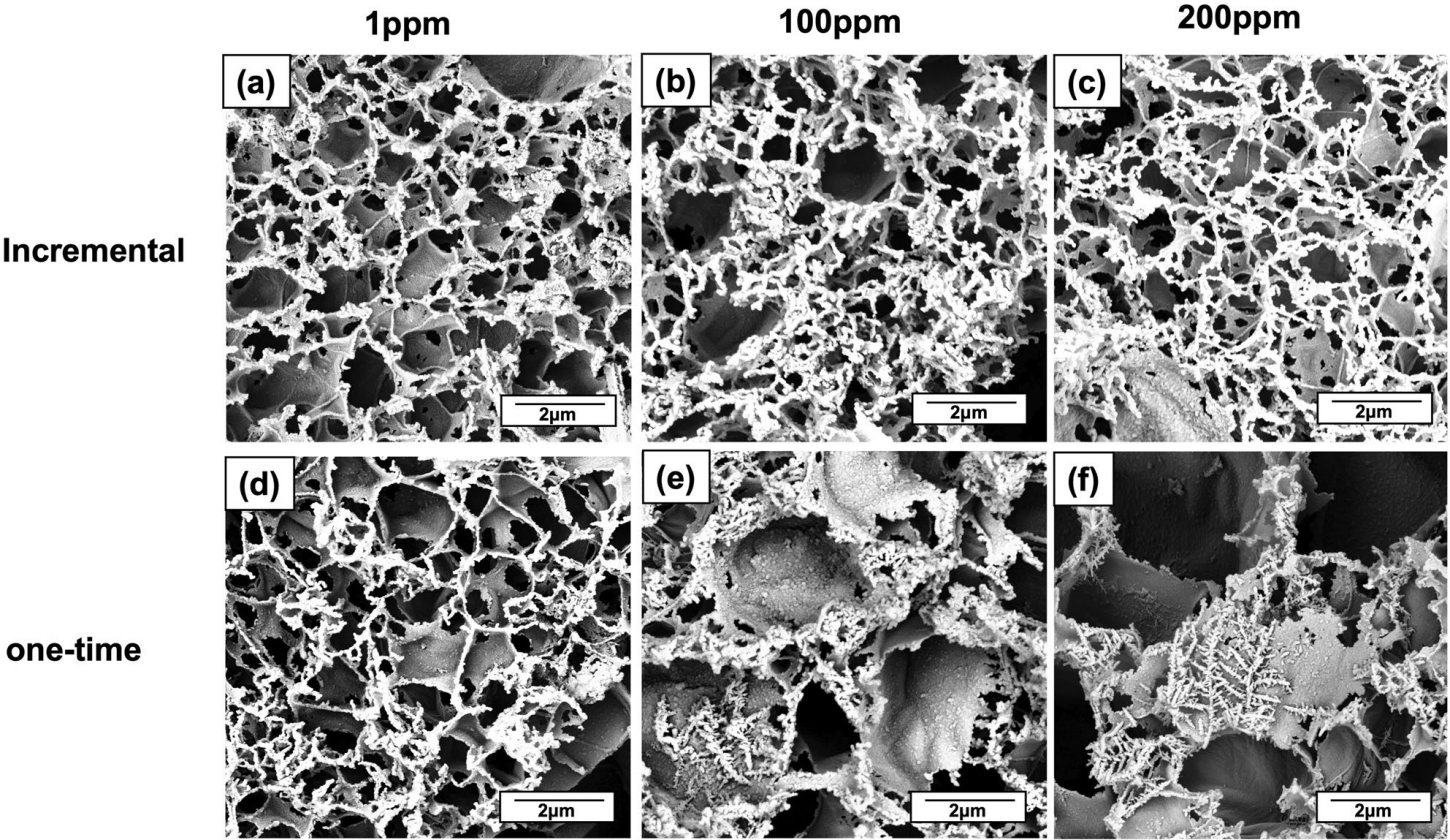
FE-SEM images of LIG surface after functionalization through one-time or incremental Al^3+^/Bi depositions. (**a)** 1 ppm of Al^3+^. (**b)** incremental up to 100 ppm of Al^3+^shows the growth of a superficial grid-like 2D network of Al^3+^/Bi, with an apparent decrease in the accessible graphene surface area. (**c)** incremental up to 200 ppm of Al^3+^ shows an increase in the number of vertices of the superficial grid-like 2D network of Al^3+^/Bi. (**d)** 1 ppm of Al^3+^ shows an equivalent surface morphology to image (**a)**. (**e)** one-time 100 ppm of Al^3+^ shows the appearance of dendritic structures nucleated from the graphene scales’ edges. (**f**)one-time 200 ppm of Al^3+^ shows a significant increase in the dendritic formations.

The calibration curve for incremental measurements is shown in Fig. 3d. In contrast to the results obtained with separate LIG-based sensors per concentration, here, the linear trend is maintained up to 100 ppm of Al^3+^ (Figure S3); after which, the signal strength decreases until a plateau is reached by 200 ppm. We believe this is because the number of available electronically active sites on the WE decrease during incremental deposition of Bi. As suggested by Baldrianova^73^, increasing the deposition time and/or Bi concentration under potentials that are less than − 0.95 V leads to a non-uniform thickness of the deposited analyte and dendritic growth. This non-uniform growth can cause high local current densities, which in turn reduce the current efficiency of Bi deposition and deposition rates^73^ and thus result in non-linear responses. This is observed in our SEM micrographs in Fig. 4. While our calibration curves confirm the reproducibility of our system, we note that variability in the bismuth deposition kinetics may impact the reproducibility of electrode measurements^85^. This variability could potentially account for any slight discrepancies in the electrode’s response observed during incremental measurements. According to Dossi et al^85^, the use carbonate solution in the desorption step described in Sect. 2.2. could potentially remove more efficiently the accumulated Bi over the WE surface, thus decrease the layer thickness.

### Morphological and chemical characterization of the modified LIG electrode

We used field emission scanning electron microscopy (FE-SEM) micrographs to evaluate the effects of the electrochemical modification on the morphology of the LIG surfaces. The analysis was performed on LIG-based electrodes used for incremental and single Al^3+^ measurements at 1, 100, and 200 ppm concentrations. In both cases, the adsorption of Bi occurred mainly at the edges of the graphene sheets, as shown in Fig. 4. This was expected, considering the number of edge defects and the presence of functional chemical groups with unpaired electrons that are highly active catalytic sites^86,87^. At 1 ppm, we observe that bismuth is deposited on the edges of LIG scales for both incremental and single-concentration measurements. However, for the incremental case, we find that the bismuth-Al^3+^ forms a lattice-like surface network with pores that are inversely proportional in size to the Al^3+^ concentration. To confirm this effect, we estimated the change in porosity from a set of FE-SEM images (Figure S6) where the porosity of the LIG surface decreased from 19.3% at 1 ppm, 13.3% at 100 ppm, and 12.1% at 200 ppm. The saturation of electronically active sites on the LIG edges (Fig. 4c) occurs past 200 ppm, hence our decision to set this upper limit.

FE-SEM micrographs for LIG surfaces with single Al^3+^ deposition at concentrations above 100 ppm led to dendritic growth (Fig. 4e and f). The formation of these structures is mainly due to non-uniform metal deposition on the LIG scale edges, which depends on the Al^3+^/Bi^3+^ exchange reaction rates, the electrolyte’s transport rate, the self-diffusion barrier for the Al^3+^/Bi clusters, and the anisotropy of these analytes. Once the edges are saturated, growth continues at the existing metalized sites instead of on the graphene-scale planes. This has already been documented for Li and Bi interacting with other metals^88–91^. The branched dendritic structures lead to a larger electrochemically-active surface area that favors an increase in electronic conductivity, which explains the linear electrochemical response beyond 100 ppm, as shown in Fig. 3b ^90^. Interestingly, the dendritic structures appear to float above the surfaces of the graphene scales, after nucleating at their edges (Figure S7). Dispersion interactions and interactions with the out-of-plane pi-orbitals in graphene could favor their parallel orientation, with respect to the graphene surface.

**Figure 5:**
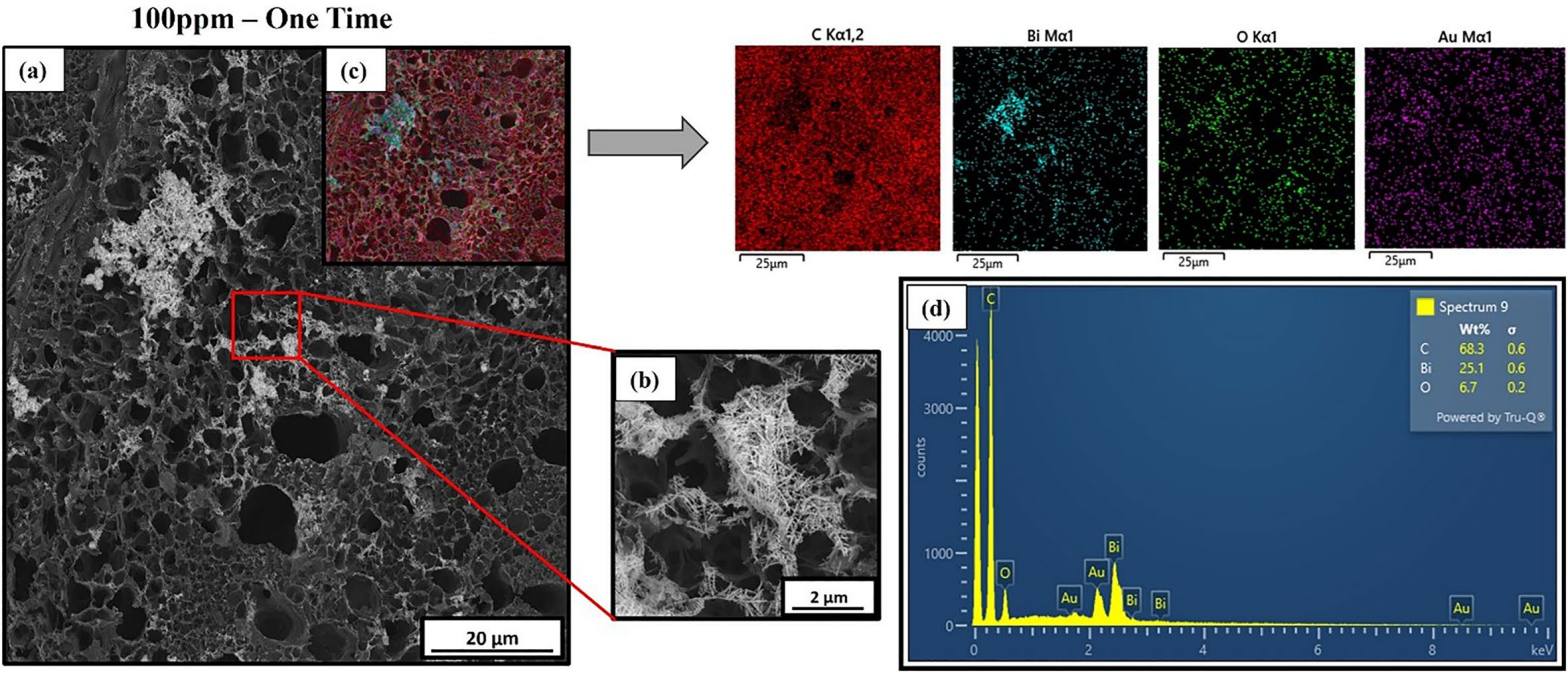
FE-SEM images of LIG surface after functionalization through one-time Al^3+^/Bi depositions. (**a)** 100 ppm of Al^3+^. (**b)** magnification the similar dendritic structure of Al^3+^/Bi grown from the edges of the graphene sheets. (**c)** EDX mapping analysis showing the distribution of the chemical elements C, O and Bi. (**d)** EDX spectrum showing the chemical composition of dendritic structure with a high content (% weight) of Bi.

The compositional analysis after functionalization was performed using a (FE-SEM), the Tescan model Clara equipped with an energy-dispersive electron X-ray (EDX) analysis system. Figure 5 shows the following images: (a) obtained at 5 kx magnification in backscattered mode to identify elements other than the electrode, (b) focused at 50 kx magnification in areas where Bi-dendritic structure growth was observed. Image (c) presents a homogeneous surface distribution of the elements carbon (C), oxygen (O), gold (Au) and bismuth (Bi) through chemical mapping analysis (EDX). EDX analysis (Fig. 5d) revealed chemical composition spectrum indicating the presence of peaks corresponding to C, O, Au and Bi. C and O are primarily in the form of graphene oxide, Au was sputtered in a thin layer (1 nm) to obtain improve image resolution, Bi is related to the electrode in-situ functionalization. Due to the low concentrations (˂ 1000 ppm) of aluminum (Al), it was not detectable using the EDX technique, which has a typical resolution limit at 0.1%. These results confirm that the functionalized solution was adsorbed on the electrode surface.

### Al^3+^ detection with our LIG-based sensor, in the presence of Cd^2+^, Pb^2+^, and Cu^2+^

To evaluate the ability of our system to detect and quantify Al^3+^ in the presence of other metal ions that are commonly found in agricultural soils, we performed negative control tests with binary and tertiary mixtures of Al^3+^, Cd^2+^, Pb^2+^, and Cu^2+^. Soils contaminated with Cd^2+^ and Pb^2+^ exhibit a sharp decrease in agricultural quality and their translocation from soil to plant tissues poses a significant threat to human health^75,92^. Despite being an essential nutrient for plants, it's important to note that copper can also be phytotoxic at high concentrations. Excessive copper levels (> 20 ppm^93^) can disrupt cellular processes, inhibit root growth, and lead to leaf chlorosis and necrosis. Copper toxicity usually occurs in acidic soils or due to excessive copper-based fungicide applications. But our intention is not to measure copper with the same electrode but to validate that it does not interfere with the oxidation potential for Bismuth in our electrochemical sensor^94^. Here, we applied SWASV on four different ion mixtures for Al^3+^, Cd^2+^, and Pb^2+^ (at 1, 100, and 200 ppm concentrations) diluted in the buffer-bismuth solution. Cu^2+^ mixtures were done at 1 and 100 ppm concentrations.

**Figure 6:**
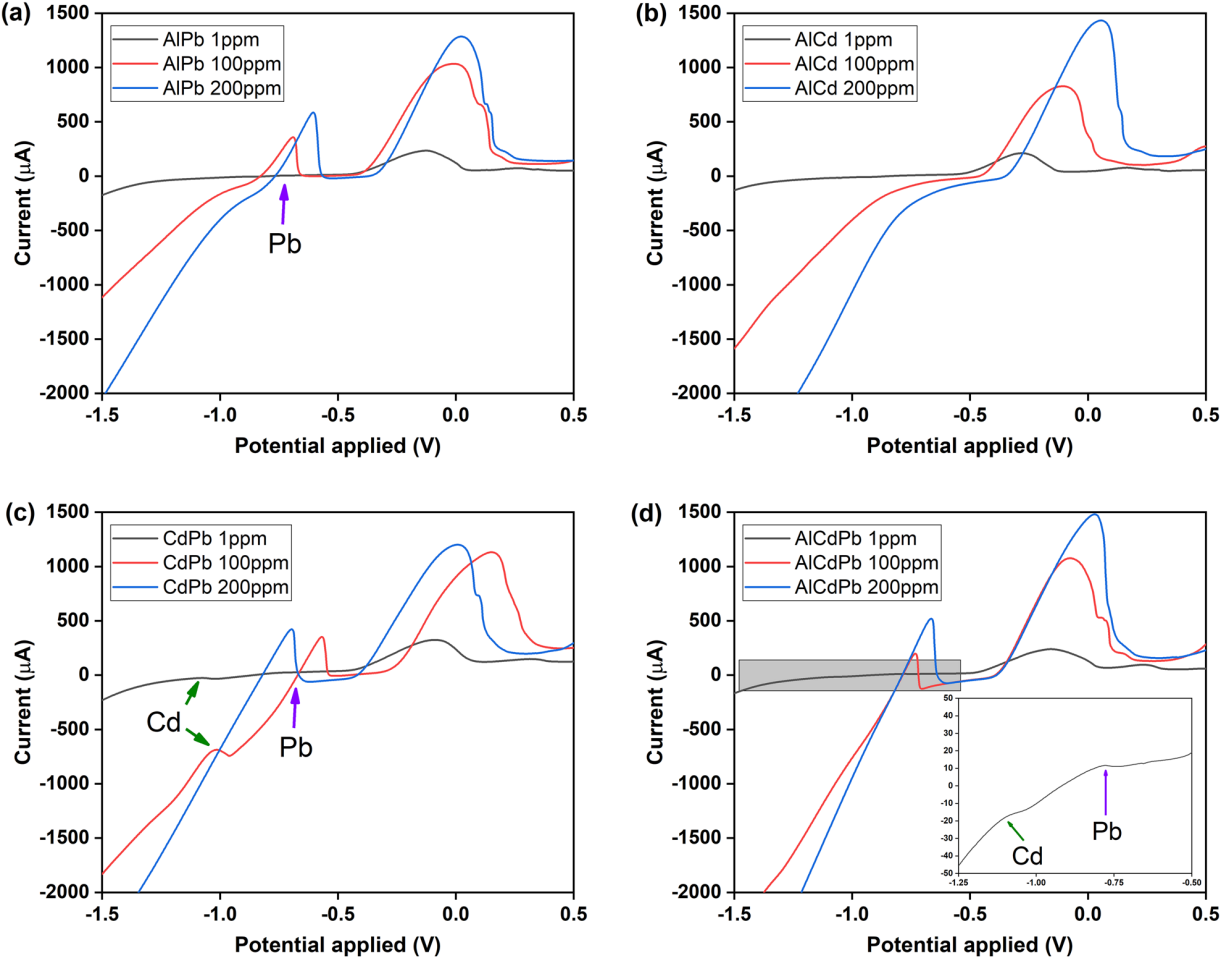
Selective detection of Al^3+^in the presence of other metallic ions. SWAVS of binary (**a**, **b**, **c**) and tertiary (**d)** mixtures of Al^3+^, Cd^2+^, and Pb^2+^.

Figure 6a shows the SWASV of Al^3+^ in the presence of Pb^2+^. The stripping peak for both ions occur at different potentials of − 0.70 V and 0.10 V for Pb^2+^ and Al^3+^, respectively. With increasing concentration, the stripping response increases for both ions. But the signal is higher for Al^3+^. In binary mixtures with Cd^2+^, only the Al^3+^-bismuth peak is observed (Fig. 6b), because Cd^2+^ requires a longer deposition time or a more negative potential to induce a stripping response at higher concentrations (Figure S8). When Cd^2+^ and Pb^2+^ were evaluated (Fig. 6c), the stripping peak for both ions was detected at − 1.00 V and − 0.70 V, respectively, consistent with the reports of simultaneous detection of these ions^25,95^. However, the Cd^2+^ signal was not detected at 200 ppm. This can be related to competition between the different analytes for the active sites on the LIG-based surface at high concentrations^95^. Figure 6d shows the case of a tertiary mixture of Al/Cd/Pb with increasing currents at different potentials, even at 1 ppm. Here, the current peak for Al^3+^ is proportional to an increase in its concentration in solution, consistent with the results shown in Fig. 3. Detailed observations of the responses are available in Figure S9. All three metal ions are selectively distinguished, albeit with a higher selectivity for Al^3+^ when the applied potential is about 0.10 V. This is consistent with other studies showing the capability of bismuth-based sensors in conjunction with an SWV method for detecting multiple ions in the same sample simultaneously^25,30,75,96^. Our results show that our LIG-based sensor has the potential to detect Cd^2+^ and Pb^2+^ heavy metal ions, which pose a significant risk to human health and can be translocated by plants from the soil to the fruits that humans subsequently consume. Nevertheless, re-optimization of the electrochemical parameters such as pH, bismuth concentration, deposition times, and others is needed^97,98^, e.g. for the detection of cadmium in cocoa plants, where the Cd concentration in the beans can range from 0.02 to 12 ppm. In comparison, the Pb concentration can be as high as 1.28 ppm, indicating the need for highly sensitive detection methods such as the electrochemical sensor developed here^98,99^.

Copper has been reported to be a common interferent in bismuth-based electrochemical systems^94,100,101^, as it affects the deposition of bismuth and consequently the stripping response of the target analyte^94^. However, no significant differences were observed in the stripping peak for Al^3+^ compared to the peak of this ion in the mixture containing Cu^2+^ at 1 ppm as shown in Figure S10 (inset). Cu is found naturally in soils, ranging from 2 to 100 ppm and averaging about 30 ppm^102,103^. At the highest Cu^2+^ concentration in soil (100 ppm), a peaked shoulder appeared on the positive side of the Al^3+^ stripping current, indicating the detection of Cu^2+^ near a potential of 0.05 V (Figure S10a). This peak does not affect the stripping signal of Al^3+^ (Figure S10b), thanks to the excess of bismuth in the system, compared to Cu^2+^ ions and the specific electrochemical conditions standardized for Al^3+^ detection. Thus, neither copper nor other ions analyzed here introduce interference in our system.

### Al^3+^ detection in solid extract samples using our LIG-based sensor 

We evaluated the performance of our system under natural conditions by quantifying the concentration of Al^3+^ via the standard addition method in four reference soils: Cauca, Carimagua, and Santander regions, and ISE-2020-3-3.1 from WEPAL. Five solutions for each sample were characterized using our LIG-based sensors, all diluted samples (1:5) with endogenous Al^3+^_,_ but at different standard concentrations (0, 10, 20, 40, and 70 ppm). We measured Al^3+^ content per sample by potentiometric titration, ICP-OES, and Flame Atomic Absortion (Table 1). Figure S11 shows the stripping voltammograms we obtained for the different Al^3+^ concentrations in each sample and their corresponding calibration curve (inset), which show an increase in the stripping current peak with increasing Al^3+^ concentration in the soil extract. We determined the Al^3+^ content in each soil sample by extrapolating to the standard addition curve (see Figure S12). Based on the Al speciation curve as a function of pH, we introduced a correction factor (Figure S5b) to account for the difference in pH between the soil and our measurement (at pH 3.70). As tabulated, the results agree well with potentiometric titration, ICP-OES, and flame atomic absorption (Table 1). This confirms that our LIG-based sensor can accurately measure Al^3+^ concentration in soil samples with a pH < 4.8, where this ion is predominant, as shown in Figure S5a.

**Table 1:** Electrochemical determination of Al^3+^ in soil extracts in comparison with standard methods.

Soil Sample	pH	EC SWASV (ppm)	EC SWASV (ppm)*	Pot. Titration (ppm)	Error %	ICP-OES (ppm)	Error %	Atomic Absorption (ppm)	Error %
Cauca	3.7	197.89	197.89	204.7	3.33	202.05	2.06	227.62	13.06
Carimagua	4.1	233.75	201.51	199.8	− 0.86	211.32	4.64	222.07	9.26
Santander	4.5	266.9	221.05	199.8	− 10.64	249.27	11.32	231.65	4.57
S-048–44	5.07	112.95	ND	34	ND	–	–	–	–
S-139–1	5.32	206.98	ND	80.1	ND	–	–	–	–
ISE-2020–3-3.1	5.4	214.95	ND	0	ND	10.86	ND	1.19	ND

ND indicates no determination.

*Corrected values according to the curve presented in Figure S5b. %Error: SWASV compared to the values obtained by each standard method. Corrected concentration was used for this aim.

The WEPAL sample gave an indeterminate value, consistent with our correction function at pH > 4.8 (Figure S5a). To corroborate this limitation, we examined two additional soil samples with intermediate and lower concentrations of Al^3+^, S-048-44 and S-139-1, at pH 5.07 and 5.32, respectively. Our corrected sensor values overestimate Al^3+^ for these cases (Table 1). There are other methods for determining the Al^3+^ concentration under these pH conditions, but they have a relatively high uncertainty due to the formation of metal complexes that bind the aluminum. This is why agricultural soils are often made alkaline; to promote metal complexes that the plant cannot absorb. Interestingly, although the activity of most Al species decreases between a pH of 4.8–5.5, phytotoxicity is still observed for some crops. Some authors suggest this is due to an increase in the Al^3+^ content in the root plasma membrane related to a dissociation of H^+^ from potentially negative ligands present in this structure during this pH range^104^. Even so, most Al^3+^ availability occurs when pH is below 5^48,104,105^, where our system and extraction protocol gives accurate results. Further optimization of the extraction procedure is needed to determine the exchangeable Al^3+^ content in soil samples within the pH range between 5 and 5.5.

## Conclusions

We demonstrate an Al^3+^ LIG-based electrochemical sensor with a linear detection range of 1.07–300 ppm for one-time measurements (single use) and 1.76–100 ppm for incremental measurements with the same electrode. This detection range is ideal for characterizing Al^3+^ concentrations in agricultural soils and determining potentially phytotoxic conditions. We show that in situ Bi modification of the WE offer an improved advantage in the fabrication protocol compared to state-of-the-art results. Another key result is that the same LIG-based Bi-functionalized sensing platform can selectively identify and measure the concentration of other metals of agronomic relevance, Cd, and Pb. Specifically, heavy metals like Cd and Pb are toxic to humans at relatively low concentrations (near the obtained LOD for our sensor). We also demonstrate that Cu, an essential element for plant growth, naturally found in soils, in some form or other, does not interfere with our sensor. Thus, the same Al sensing platform can determine toxic-to-human levels of Cd and Pb and phytotoxic levels of Al^3+^. Increasing the sensitivity for Cd and Pb would only require an adjustment in the Bi concentration and pH during the fabrication of the sensor. The low cost, ease of use, and straightforward scalability of our sensor are all factors that underscore its potential to improve agricultural practices, including performing and tracking soil amendments (e.g., liming) or monitoring phytoremediation strategies, such as using aluminum hyper-accumulating plants by determining the Al^3+^ content in its tissues.

### Supplementary Information

Supplementary Figures.

## Data Availability

The Supporting Information is available free of charge on the Journal’s website. Supplementary information Al^3+^.pdf. The datasets generated during and/or analyzed during the current study are available from the corresponding author on reasonable request.
