# Supplementary material for: Laser-induced graphene electrochemical sensor for quantitative detection of phytotoxic aluminum ions (Al3+) in soils extracts
Source: Sci Rep. 2024 Mar 8;14:5772. doi: 10.1038/s41598-024-56212-0 (PMC10923804; doi:10.1038/s41598-024-56212-0)
Supplement: Supplementary file 1 — Supplementary Figures. [file 41598_2024_56212_MOESM1_ESM.docx]

**Supplementary information Laser-Induced Graphene Electrochemical Sensor for Quantitative Detection of Phytotoxic Aluminum Ions (Al^3+^) in Soils Extracts**

Vanessa Reyes-Loaiza^a^, Jhonattan De La Roche^a^, Erick Hernandez Renjifo^a^, Orlando Idárraga^b,c^, Mayesse Da Silva^b^, Drochss P. Valencia^a^, Thaura Ghneim-Herrera^d,a^ and Andres Jaramillo-Botero^e,a^*

^a^ Omicas Alliance, Pontificia Universidad Javeriana, Cali, Valle del Cauca 760031, Colombia

^b^ Multifunctional landscapes, Alliance Bioversity-CIAT, Cali-Palmira, Valle del Cauca 763537, Colombia

^c^ Department of Natural and Exact Sciences, Universidad del Valle, Cali, Valle del Cauca 760031, Colombia

^d^ Department of Biological Sciences, Universidad ICESI, Cali, Valle del Cauca 760031, Colombia

^e^ Chemistry and Chemical Engineering Division, California Institute of Technology, 1200 E California Blvd, Pasadena, CA 91125, USA.


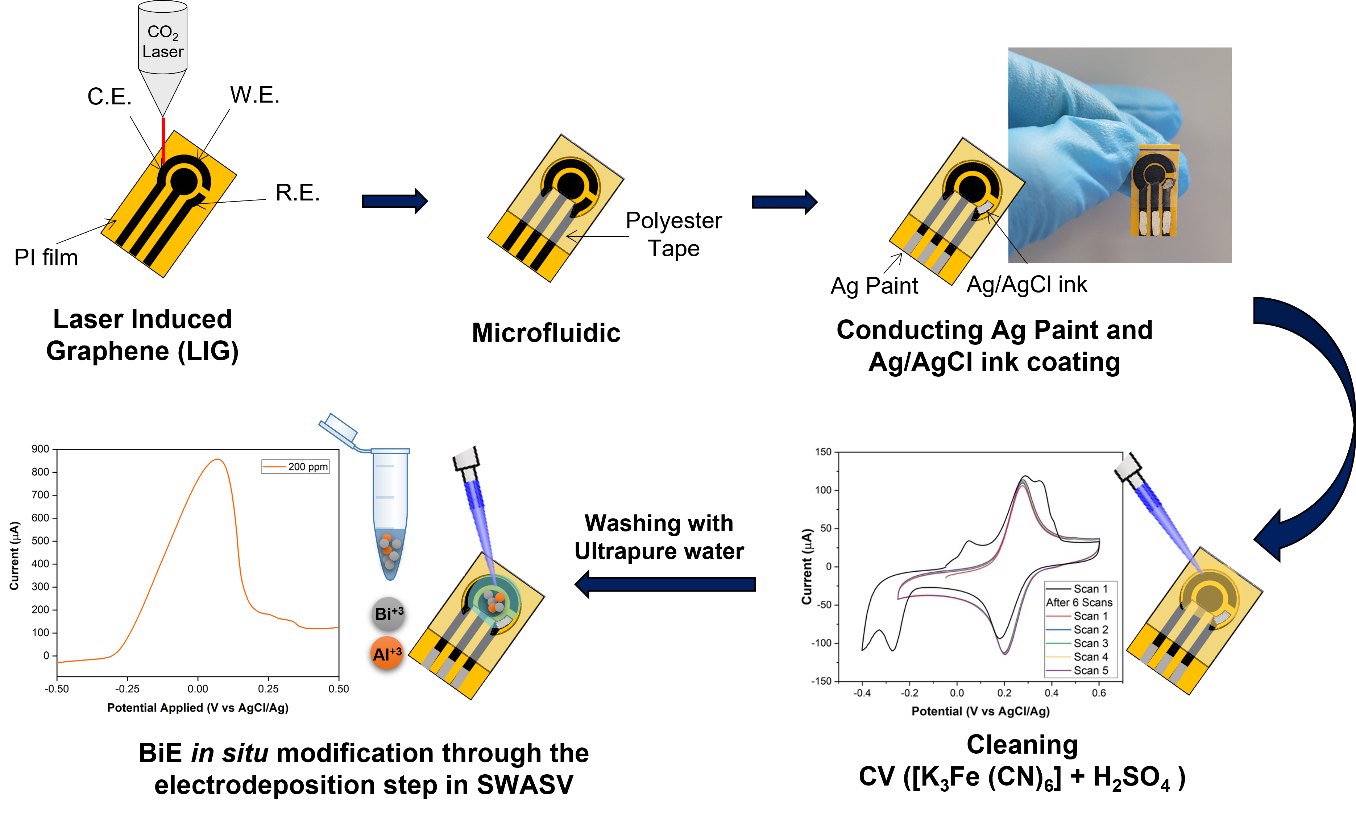


Figure S1. Schematic process of BiE-LIG electrode fabrication.


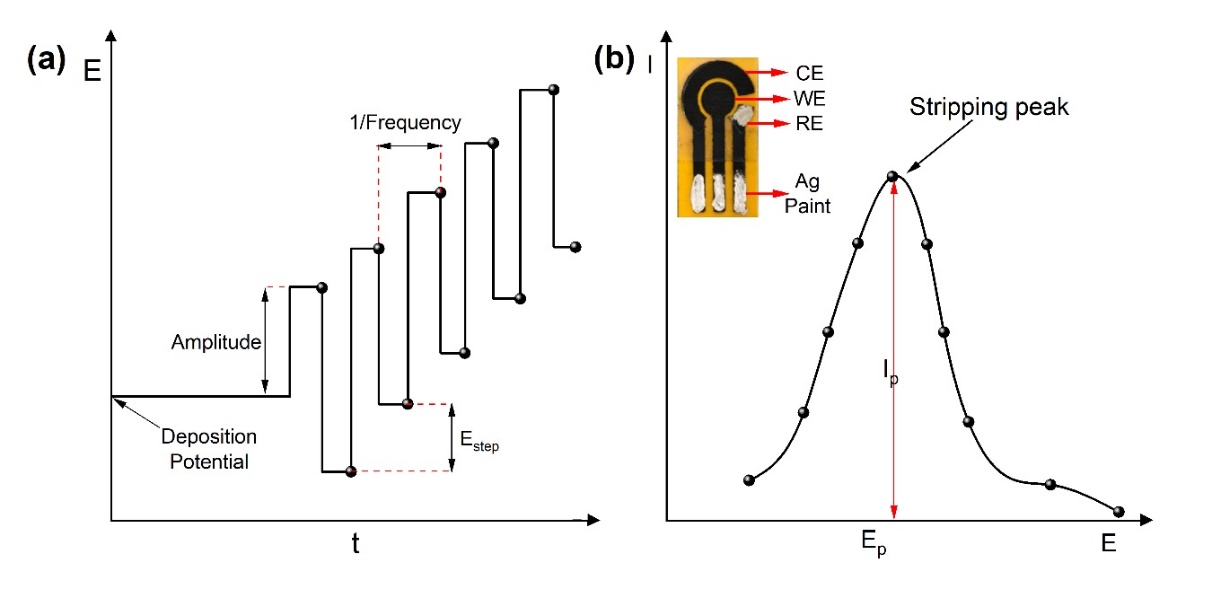


Figure S2. a) General waveform for the Square Wave Anodic Stripping Voltammetry (SWAVS) used in this work. b) Stripping peak in the IV characteristic response to the SWAVS. Inset shows the picture of our LIG electrochemical cell design, based on three interdigitated electrodes, namely a working electrode (WE), a counter electrode (CE), and an Ag/AgCl casted pseudo reference electrode (RE), and the Ag-coated contacts, over a Polyimide tape substrate. Figures were adapted and modified from [1,2]

S. E. Jeong, S. Kim, J. H. Han, and J. J. Pak, “Simple laser-induced graphene fiber electrode fabrication for high-performance heavy-metal sensing,” *Microchem. J.*, vol. 172, no. PA, p. 106950, 2022.

G. Zhao and G. Liu, “A portable electrochemical system for the on-site detection of heavy metals in farmland soil based on electrochemical sensors,” *IEEE Sens. J.*, vol. 18, no. 14, pp. 5645–5655, 2018.

Figure S3. Calibration curve for incremental measurements for Al^3+^ from 10 to 100 ppm.


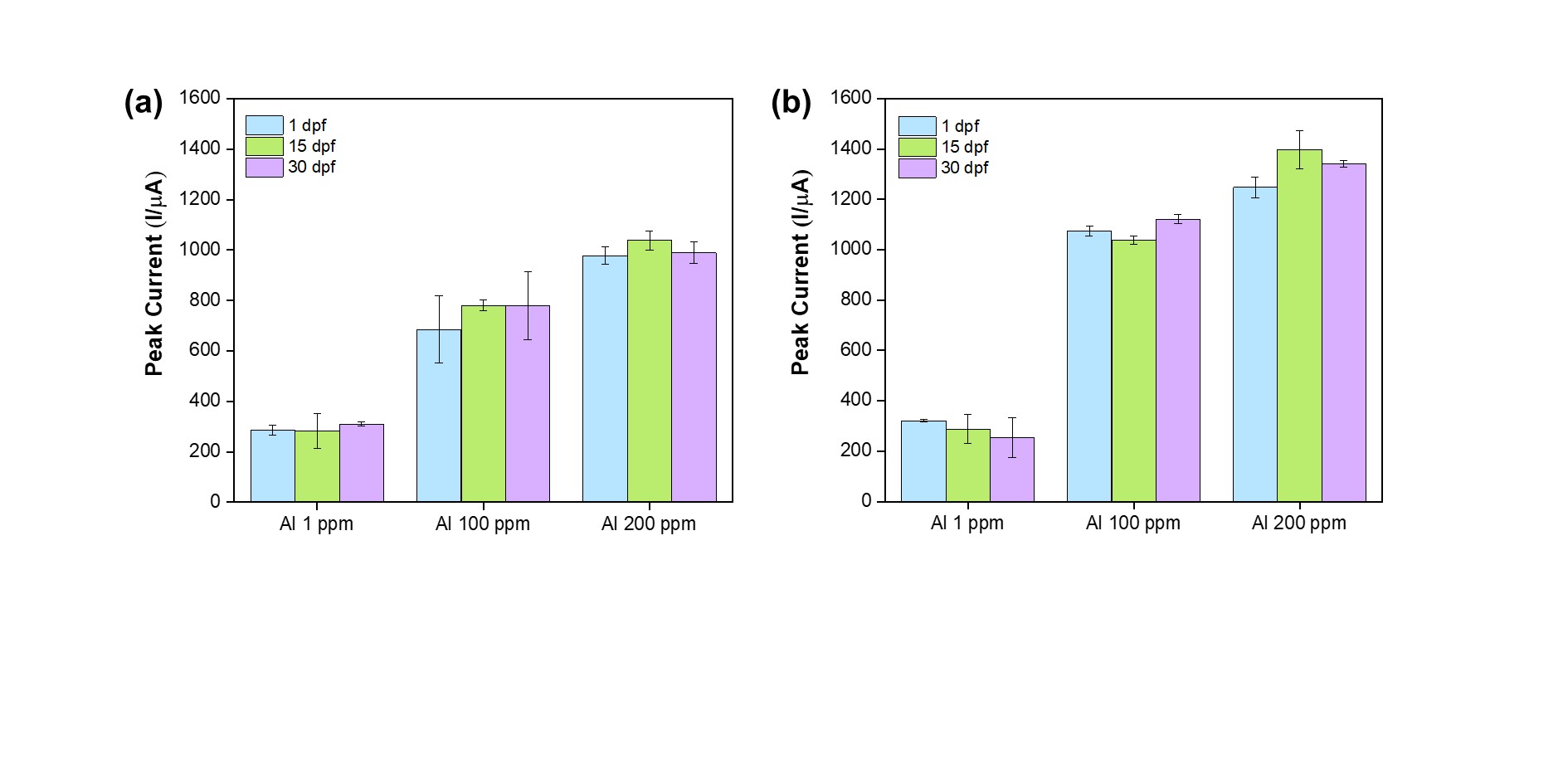


Figure S4. Stability test of the electrochemical sensor’s response to Al^3+^. Comparison of the peak current response of electrodes with the longevity of 1, 15, and 30 days post fabrication (dpf) at three concentrations of Al3^+^ 1, 100, and 200 ppm. In a) One-time measurements and b) incremental measurements.


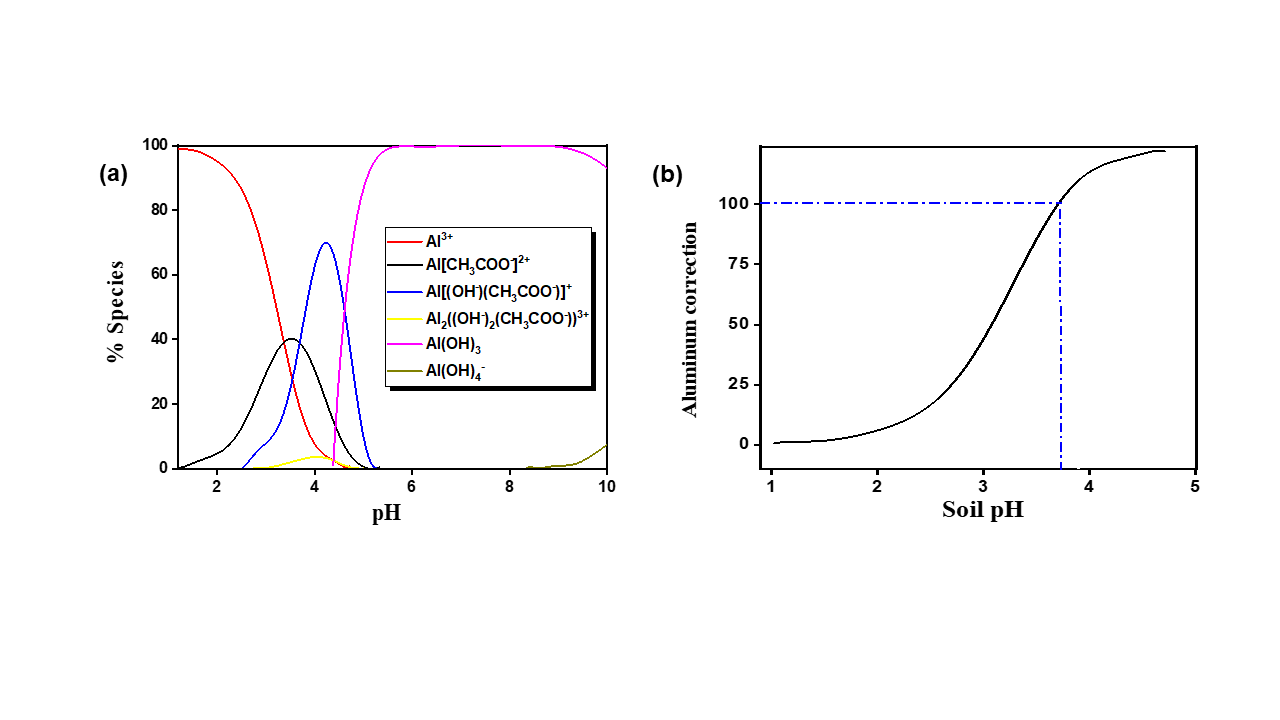


Figure S5. a) Percentage distribution of aluminum species in an acetate solution relative to pH. b) Correction curve for the determination of Al3+ at pH 3.7 as a function of soil pH. To use it, any value obtained at 3.7 is multiplied by 100 and divides by the correction percentage by extrapolation dependent on soil pH. The dotted blue line indicates pH 3.7.


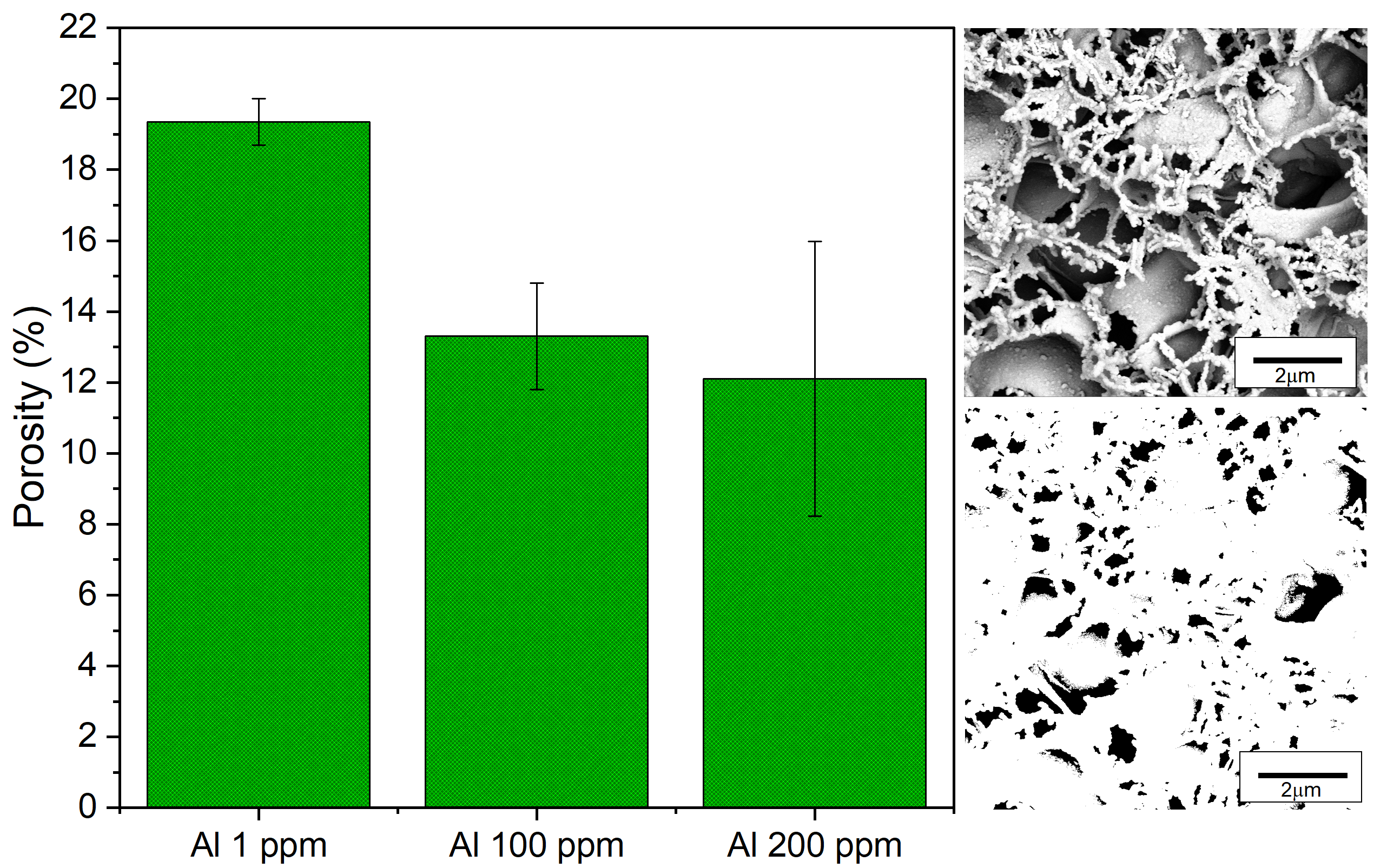


Figure S6. Porosity results from the analysis of the Bismuth network deposited into graphene edges. Images show the FE-SEM and the binarization using ImageJ.


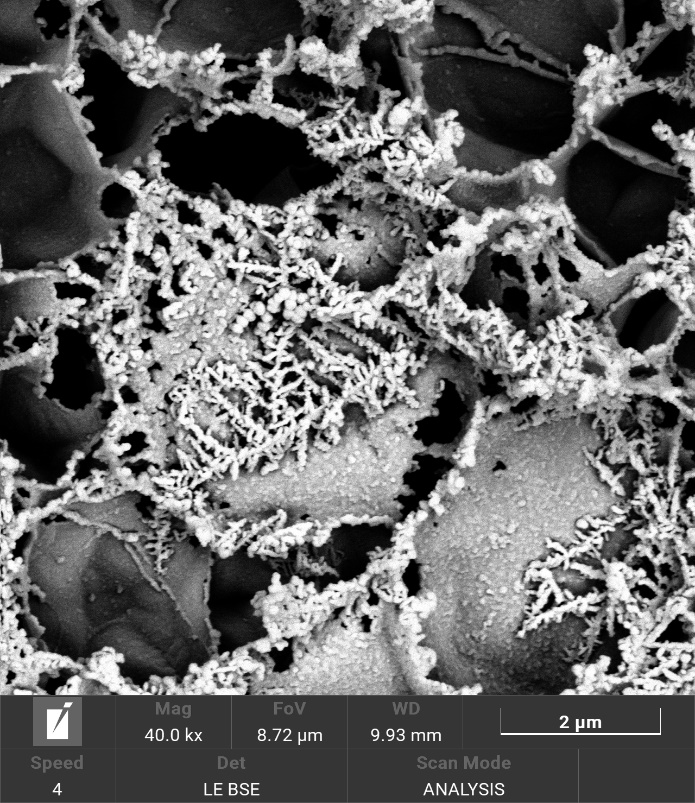


Figure S7. FE-SEM image of the dendritic growth with a concentration Al^3+^ of 200 ppm. Nucleation initiates at the edges and extents over graphene surface.


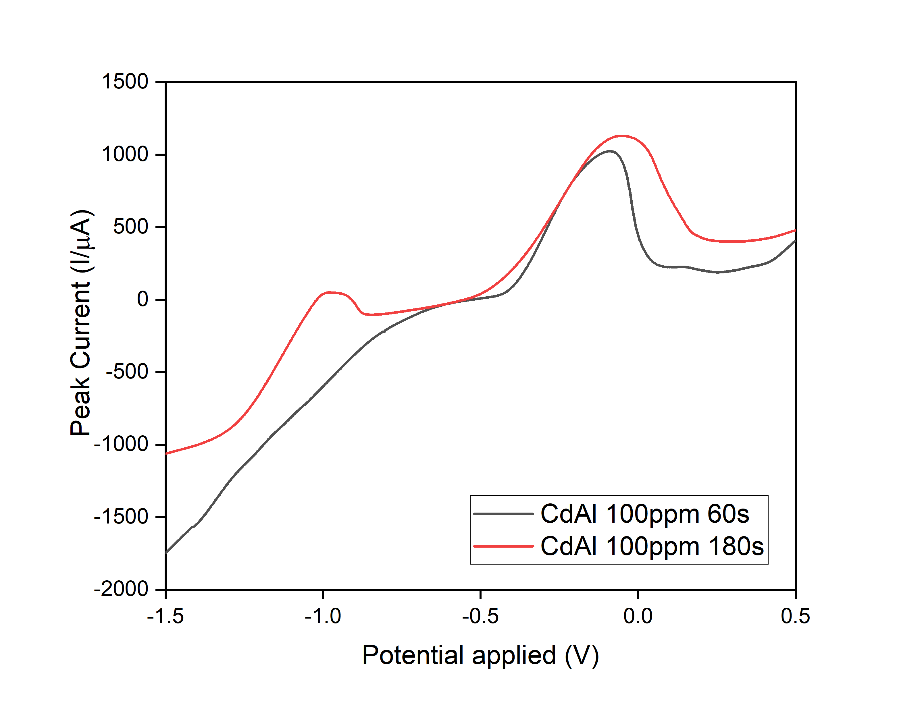


Figure S8. Influence of the deposition time in the peak current for simultaneous detection of Cadmium and Aluminum ions in an acetate buffer-bismuth solution. The experiment involved a mixture of each ion at a concentration of 100 ppm.


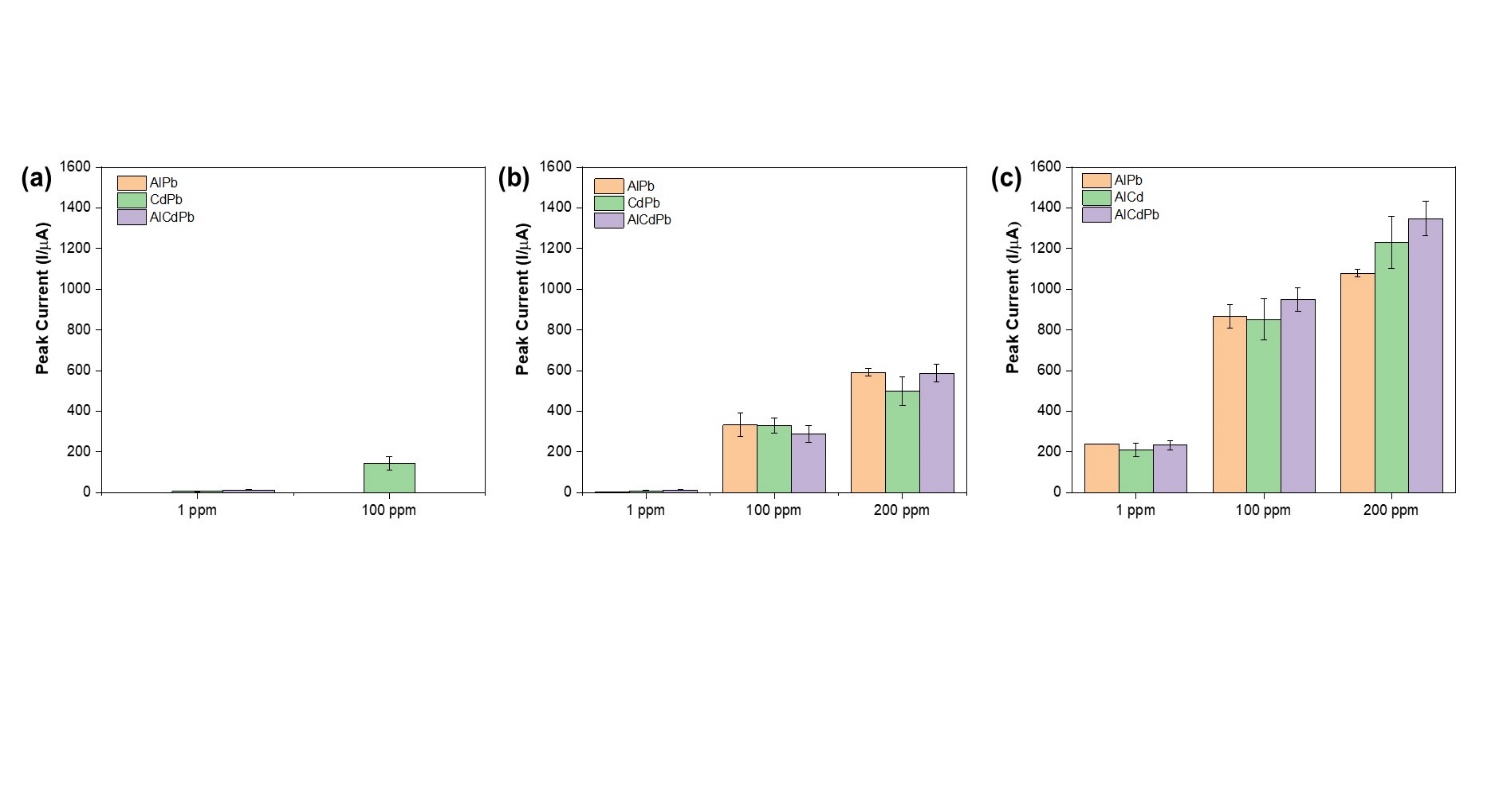


Figure S9. Peak current of each ion a) Cd2+, b) Pb2+ and c) Al3+ in binary and tertiary mixtures of Al^3+^, Cd^2+^, and Pb^2+^.


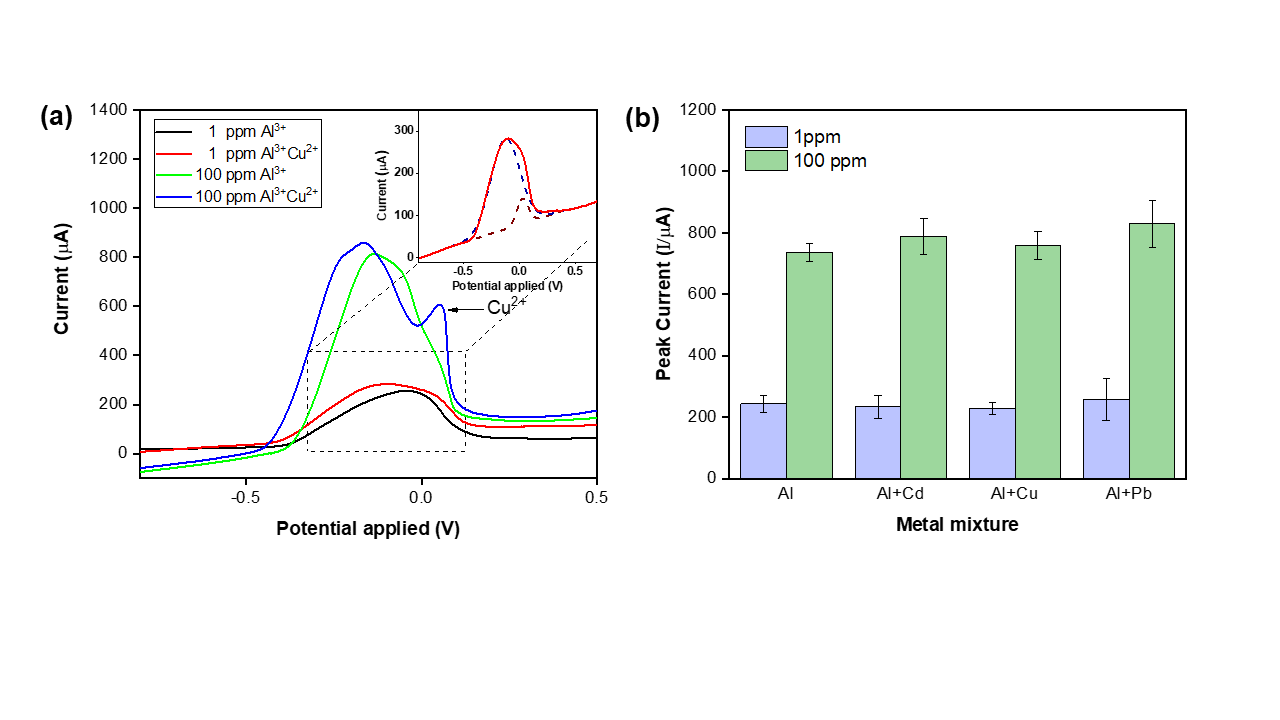


Figure S10. Quantification of Al^3+^ in the presence of other metal ions at a low (1ppm) and a high concentration (100ppm). a) SWASV of Al^3+^ in the presence of Cu^2+^. Inset shows the deconvolution of the stripping response for the Al^3+^ and Cu^2+^ mixture, individual peaks for each ion are represented in the dashed lines, blue for Al^3+^ and dark red for Cu^2+^. b) Comparison of the peak current of Al^3+^ alone in solution and in the presence of other metal ions.





Figure S11. Stripping voltammograms for the detection of Al^3+^ in soil samples from regions in Colombia a) Cauca b) Carimagua c) Santander and d) Reference soil sample ISE-2020-3-3.1 (WEPAL). Inset shows the calibration curve obtained from the standard addition method (data points correspond to mean and standard deviation of three replicates).





Figure S12. Standard addition curve for Al^3+^ detection in soil extract. Aluminum concentration in the sample is determined when peak current=0.
